# 3-[1-(3-Hydroxy­benz­yl)-1*H*-benz­imid­azol-2-yl]phenol

**DOI:** 10.1107/S1600536809018698

**Published:** 2009-05-23

**Authors:** Naser Eltaher Eltayeb, Siang Guan Teoh, Hoong-Kun Fun, Samuel Robinson Jebas, Rohana Adnan

**Affiliations:** a School of Chemical Science, Universiti Sains Malaysia, Minden, Penang, Malaysia; b X-ray Crystallography Unit, School of Physics, Universiti Sains Malaysia, 11800 USM, Penang, Malaysia

## Abstract

In the title mol­ecule, C_20_H_16_N_2_O_2_, the benzimidazole mean plane forms dihedral angles of 56.55 (3) and 81.65 (4)° with the two benzene rings. In the crystal structure, inter­molecular O—H⋯O and O—H⋯N hydrogen bonds link the mol­ecules into layers parallel to the (101) plane. The crystal packing also exhibits weak inter­molecular C—H⋯O and C—H⋯π inter­actions.

## Related literature

For the biological activity of benzimidazole derivatives, see: Demirayak *et al.* (2002 ▶); Minoura *et al.* (2004 ▶); Pawar *et al.* (2004 ▶); Tomei *et al.* (2003 ▶). For related structures, see: Eltayeb *et al.* (2007*a* ▶,*b* ▶,*c* ▶). For bond-length data, see: Allen *et al.* (1987 ▶). For the stability of the temperature controller used in the data collection, see: Cosier & Glazer (1986 ▶).
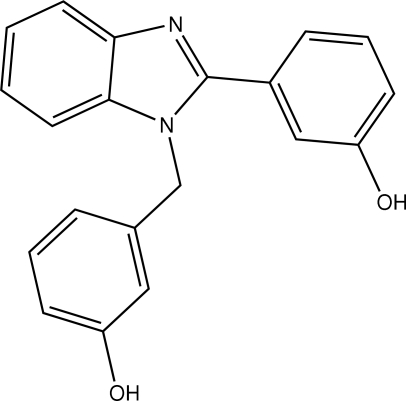


## Experimental

### 

#### Crystal data

C_20_H_16_N_2_O_2_
*M* *_r_* = 316.35
Monoclinic, 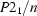

*a* = 10.5128 (2) Å
*b* = 12.1096 (2) Å
*c* = 12.5235 (2) Å
β = 96.948 (1)°
*V* = 1582.61 (5) Å^3^
*Z* = 4
Mo *K*α radiation
μ = 0.09 mm^−1^
*T* = 100 K
0.55 × 0.34 × 0.15 mm

#### Data collection

Bruker SMART APEXII CCD area-detector diffractometer
Absorption correction: multi-scan (*SADABS*; Bruker, 2005 ▶) *T*_min_ = 0.954, *T*_max_ = 0.987
34106 measured reflections
7426 independent reflections
6004 reflections with *I* > 2σ(*I*)
*R*_int_ = 0.040

#### Refinement

*R*[*F* ^2^ > 2σ(*F* ^2^)] = 0.048
*wR*(*F* ^2^) = 0.136
*S* = 1.05
7426 reflections
225 parameters
H atoms treated by a mixture of independent and constrained refinement
Δρ_max_ = 0.51 e Å^−3^
Δρ_min_ = −0.29 e Å^−3^

### 

Data collection: *APEX2* (Bruker, 2005 ▶); cell refinement: *SAINT* (Bruker, 2005 ▶); data reduction: *SAINT*; program(s) used to solve structure: *SHELXTL* (Sheldrick, 2008 ▶); program(s) used to refine structure: *SHELXTL*; molecular graphics: *SHELXTL*; software used to prepare material for publication: *SHELXTL* and *PLATON* (Spek, 2009 ▶).

## Supplementary Material

Crystal structure: contains datablocks global, I. DOI: 10.1107/S1600536809018698/cv2563sup1.cif

Structure factors: contains datablocks I. DOI: 10.1107/S1600536809018698/cv2563Isup2.hkl

Additional supplementary materials:  crystallographic information; 3D view; checkCIF report

## Figures and Tables

**Table 1:** Hydrogen-bond geometry (Å, °)

*D*—H⋯*A*	*D*—H	H⋯*A*	*D*⋯*A*	*D*—H⋯*A*
O1—H1O1⋯O2^i^	0.931 (19)	1.712 (19)	2.6406 (9)	175.4 (17)
O2—H1O2⋯N2^ii^	0.994 (19)	1.646 (19)	2.6297 (10)	169.7 (16)
C3—H3*A*⋯O1^iii^	0.93	2.57	3.2987 (10)	136
C9—H9*A*⋯O1^iv^	0.93	2.59	3.4287 (10)	150
C12—H12*A*⋯*Cg*1^ii^	0.93	2.67	3.4521 (9)	142

Symmetry codes: (i) 
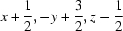
; (ii) 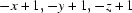; (iii) 
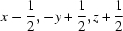
; (iv) 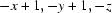. *Cg*1 is the centroid of the ring C1–C6.

**Fig. 1.**
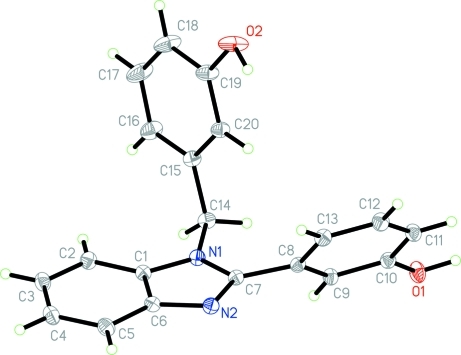
The molecular structure of (I), showing 50% probability displacement ellipsoids and the atom numbering scheme.

## supplementary crystallographic information

### Comment

The synthesis of benzimidazoles has received much attention owing to the varied biological activities such as antidiabetic (Minoura *et al.*, 2004), antimicrobial, antifungal (Pawar *et al.*, 2004), antiviral (Tomei *et al.*, 2003), and anticancer (Demirayak *et al.*, 2002) properties exhibited by a number of derivatives of these compounds. In continuation of our structural study of benzimidazole derivatives (Eltayeb *et al.* 2007*a*,*b*,*c*), we describe in this paper the crystal structure of the title compound (I).

In (I) (Fig. 1), the bond lengths and bond angles are normal (Allen *et al.*, 1987). The benzimidazole unit is planar with the maximum deviation from planarity of 0.0403 (9) Å for atom C3. The dihedral angle formed by the benzimidazole unit with the two benzene rings (C8–C13 and C15–C20) are 56.55 (3)° and 81.65 (4)° respectively. The two benzene rings (C8–C13 and C15–C20) are inclined to each other forming a dihedral angle of 72.54 (4)°.

In the crystal, intermolecular O—H···O, O—H···N hydrogen bonds (Table 1) link the molecules into layers parallel to the (101) plane. The crystal packing exhibits also weak C—H···O and C—H···π interactions (Table 1).

### Experimental

To a solution of *o*-phenylenediamine (0.216 g, 2 mmol) in ethanol (30 ml) was added 3-hydroxybenzaldehyde (0.488 g, 4 mmol). The mixture was refluxed with stirring for half an hour. The resultant yellow solution was filtered. Crystals suitable for XRD were formed after several days of slow evaporation of solvent at room temperature.

### Refinement

H atoms were positioned geometrically [C—H = 0.93–0.97 Å] and refined using a riding model with *U*_iso_(H) = 1.2*U*_eq_(C). The O-bound H atoms were located on a Fourier map and were refined isotropically.

### Crystal data

**Table:**

C_20_H_16_N_2_O_2_	*F*(000) = 664
*M**_r_* = 316.35	*D*_x_ = 1.328 Mg m^−^^3^
Monoclinic, *P*2_1_/*n*	Mo *K*α radiation, λ = 0.71073 Å
Hall symbol: -P 2yn	Cell parameters from 8327 reflections
*a* = 10.5128 (2) Å	θ = 2.4–37.2°
*b* = 12.1096 (2) Å	µ = 0.09 mm^−^^1^
*c* = 12.5235 (2) Å	*T* = 100 K
β = 96.948 (1)°	Plate, colourless
*V* = 1582.61 (5) Å^3^	0.55 × 0.34 × 0.15 mm
*Z* = 4	

### Data collection

**Table:**

Bruker SMART APEXII CCD area-detector diffractometer	7426 independent reflections
Radiation source: fine-focus sealed tube	6004 reflections with *I* > 2σ(*I*)
graphite	*R*_int_ = 0.040
φ and ω scans	θ_max_ = 36.0°, θ_min_ = 2.4°
Absorption correction: multi-scan (*SADABS*; Bruker, 2005)	*h* = −14→17
*T*_min_ = 0.954, *T*_max_ = 0.987	*k* = −20→20
34106 measured reflections	*l* = −19→20

### Refinement

**Table:**

Refinement on *F*^2^	Primary atom site location: structure-invariant direct methods
Least-squares matrix: full	Secondary atom site location: difference Fourier map
*R*[*F*^2^ > 2σ(*F*^2^)] = 0.048	Hydrogen site location: inferred from neighbouring sites
*wR*(*F*^2^) = 0.136	H atoms treated by a mixture of independent and constrained refinement
*S* = 1.05	*w* = 1/[σ^2^(*F*_o_^2^) + (0.0723*P*)^2^ + 0.2344*P*] where *P* = (*F*_o_^2^ + 2*F*_c_^2^)/3
7426 reflections	(Δ/σ)_max_ < 0.001
225 parameters	Δρ_max_ = 0.51 e Å^−^^3^
0 restraints	Δρ_min_ = −0.29 e Å^−^^3^

### Special details

**Table:**

Experimental. The crystal was placed in the cold stream of an Oxford Cyrosystems Cobra open-flow nitrogen cryostat (Cosier & Glazer, 1986) operating at 100.0 (1) K.
Geometry. All e.s.d.'s (except the e.s.d. in the dihedral angle between two l.s. planes) are estimated using the full covariance matrix. The cell e.s.d.'s are taken into account individually in the estimation of e.s.d.'s in distances, angles and torsion angles; correlations between e.s.d.'s in cell parameters are only used when they are defined by crystal symmetry. An approximate (isotropic) treatment of cell e.s.d.'s is used for estimating e.s.d.'s involving l.s. planes.
Refinement. Refinement of *F*^2^ against ALL reflections. The weighted *R*-factor *wR* and goodness of fit *S* are based on *F*^2^, conventional *R*-factors *R* are based on *F*, with *F* set to zero for negative *F*^2^. The threshold expression of *F*^2^ > σ(*F*^2^) is used only for calculating *R*-factors(gt) *etc*. and is not relevant to the choice of reflections for refinement. *R*-factors based on *F*^2^ are statistically about twice as large as those based on *F*, and *R*- factors based on ALL data will be even larger.

### Fractional atomic coordinates and isotropic or equivalent isotropic displacement parameters (Å^2^)

**Table:**

	*x*	*y*	*z*	*U*_iso_*/*U*_eq_	
O1	0.60369 (7)	0.60548 (5)	0.04595 (5)	0.01932 (13)	
O2	0.21201 (7)	0.70928 (6)	0.49193 (6)	0.02850 (16)	
N1	0.36876 (7)	0.35014 (5)	0.31082 (5)	0.01366 (12)	
N2	0.55084 (7)	0.30903 (6)	0.41524 (5)	0.01468 (12)	
C1	0.34113 (8)	0.26119 (6)	0.37376 (6)	0.01385 (13)	
C2	0.22644 (8)	0.20556 (7)	0.38301 (6)	0.01625 (14)	
H2A	0.1502	0.2247	0.3415	0.019*	
C3	0.23254 (9)	0.12006 (7)	0.45759 (7)	0.01787 (15)	
H3A	0.1581	0.0820	0.4676	0.021*	
C4	0.34872 (9)	0.08978 (7)	0.51829 (7)	0.01903 (16)	
H4A	0.3497	0.0308	0.5659	0.023*	
C5	0.46206 (9)	0.14602 (7)	0.50872 (7)	0.01765 (15)	
H5A	0.5388	0.1255	0.5486	0.021*	
C6	0.45615 (8)	0.23468 (6)	0.43671 (6)	0.01408 (13)	
C7	0.49493 (8)	0.37658 (6)	0.34028 (6)	0.01328 (13)	
C8	0.55758 (8)	0.47449 (6)	0.29966 (6)	0.01346 (13)	
C9	0.55681 (8)	0.49315 (6)	0.18938 (6)	0.01442 (13)	
H9A	0.5214	0.4411	0.1398	0.017*	
C10	0.60956 (8)	0.59058 (6)	0.15413 (6)	0.01466 (14)	
C11	0.66423 (8)	0.66833 (7)	0.22849 (6)	0.01679 (15)	
H11A	0.6988	0.7333	0.2049	0.020*	
C12	0.66666 (9)	0.64783 (7)	0.33810 (7)	0.01769 (15)	
H12A	0.7041	0.6990	0.3876	0.021*	
C13	0.61356 (8)	0.55155 (7)	0.37433 (6)	0.01600 (14)	
H13A	0.6153	0.5385	0.4477	0.019*	
C14	0.27189 (8)	0.40916 (7)	0.23858 (6)	0.01493 (14)	
H14A	0.2199	0.3560	0.1948	0.018*	
H14B	0.3144	0.4556	0.1907	0.018*	
C15	0.18581 (8)	0.47993 (7)	0.29866 (6)	0.01546 (14)	
C16	0.05416 (9)	0.46392 (8)	0.28541 (7)	0.02214 (17)	
H16A	0.0182	0.4078	0.2409	0.027*	
C17	−0.02425 (10)	0.53232 (10)	0.33909 (9)	0.0298 (2)	
H17A	−0.1126	0.5226	0.3289	0.036*	
C18	0.02893 (10)	0.61473 (9)	0.40753 (8)	0.0276 (2)	
H18A	−0.0235	0.6601	0.4432	0.033*	
C19	0.16150 (9)	0.62907 (8)	0.42254 (7)	0.02011 (16)	
C20	0.23951 (8)	0.56315 (7)	0.36681 (6)	0.01647 (14)	
H20A	0.3276	0.5746	0.3750	0.020*	
H1O1	0.6430 (17)	0.6716 (15)	0.0307 (14)	0.053 (5)*	
H1O2	0.3026 (18)	0.6952 (15)	0.5216 (14)	0.055 (5)*	

### Atomic displacement parameters (Å^2^)

**Table:**

	*U*^11^	*U*^22^	*U*^33^	*U*^12^	*U*^13^	*U*^23^
O1	0.0266 (3)	0.0181 (3)	0.0133 (2)	−0.0054 (2)	0.0028 (2)	0.00132 (19)
O2	0.0206 (3)	0.0285 (3)	0.0336 (4)	0.0103 (3)	−0.0083 (3)	−0.0167 (3)
N1	0.0134 (3)	0.0145 (3)	0.0127 (3)	0.0004 (2)	0.0002 (2)	0.0011 (2)
N2	0.0139 (3)	0.0155 (3)	0.0144 (3)	0.0006 (2)	0.0007 (2)	0.0009 (2)
C1	0.0148 (3)	0.0135 (3)	0.0131 (3)	−0.0001 (2)	0.0012 (3)	−0.0002 (2)
C2	0.0151 (3)	0.0170 (3)	0.0162 (3)	−0.0022 (3)	0.0003 (3)	−0.0013 (2)
C3	0.0204 (4)	0.0161 (3)	0.0173 (3)	−0.0041 (3)	0.0029 (3)	−0.0011 (2)
C4	0.0242 (4)	0.0149 (3)	0.0181 (3)	−0.0023 (3)	0.0027 (3)	0.0015 (3)
C5	0.0197 (4)	0.0158 (3)	0.0169 (3)	0.0008 (3)	0.0001 (3)	0.0024 (2)
C6	0.0147 (3)	0.0136 (3)	0.0137 (3)	0.0002 (2)	0.0008 (3)	−0.0003 (2)
C7	0.0131 (3)	0.0143 (3)	0.0124 (3)	0.0001 (2)	0.0013 (2)	−0.0007 (2)
C8	0.0122 (3)	0.0142 (3)	0.0140 (3)	0.0001 (2)	0.0018 (2)	−0.0003 (2)
C9	0.0159 (3)	0.0142 (3)	0.0131 (3)	−0.0019 (2)	0.0018 (3)	−0.0008 (2)
C10	0.0154 (3)	0.0151 (3)	0.0138 (3)	−0.0006 (3)	0.0026 (3)	−0.0001 (2)
C11	0.0183 (4)	0.0148 (3)	0.0173 (3)	−0.0026 (3)	0.0023 (3)	−0.0013 (2)
C12	0.0186 (4)	0.0180 (3)	0.0165 (3)	−0.0035 (3)	0.0021 (3)	−0.0036 (2)
C13	0.0161 (4)	0.0180 (3)	0.0138 (3)	−0.0011 (3)	0.0016 (3)	−0.0019 (2)
C14	0.0145 (3)	0.0177 (3)	0.0120 (3)	0.0020 (3)	−0.0010 (3)	0.0002 (2)
C15	0.0148 (3)	0.0176 (3)	0.0136 (3)	0.0022 (3)	0.0000 (3)	0.0002 (2)
C16	0.0145 (4)	0.0283 (4)	0.0229 (4)	0.0005 (3)	−0.0007 (3)	−0.0069 (3)
C17	0.0133 (4)	0.0409 (6)	0.0349 (5)	0.0020 (4)	0.0017 (4)	−0.0144 (4)
C18	0.0162 (4)	0.0369 (5)	0.0292 (5)	0.0071 (4)	0.0002 (3)	−0.0126 (4)
C19	0.0174 (4)	0.0217 (4)	0.0200 (4)	0.0057 (3)	−0.0028 (3)	−0.0053 (3)
C20	0.0137 (3)	0.0178 (3)	0.0172 (3)	0.0026 (3)	−0.0006 (3)	−0.0017 (3)

### Geometric parameters (Å, °)

**Table:**

O1—C10	1.3607 (10)	C9—C10	1.3977 (11)
O1—H1O1	0.931 (19)	C9—H9A	0.9300
O2—C19	1.3671 (11)	C10—C11	1.3980 (11)
O2—H1O2	0.994 (19)	C11—C12	1.3922 (11)
N1—C7	1.3709 (10)	C11—H11A	0.9300
N1—C1	1.3861 (10)	C12—C13	1.3922 (11)
N1—C14	1.4641 (11)	C12—H12A	0.9300
N2—C7	1.3275 (10)	C13—H13A	0.9300
N2—C6	1.3923 (10)	C14—C15	1.5114 (11)
C1—C2	1.3981 (11)	C14—H14A	0.9700
C1—C6	1.3986 (12)	C14—H14B	0.9700
C2—C3	1.3906 (11)	C15—C16	1.3874 (13)
C2—H2A	0.9300	C15—C20	1.3951 (11)
C3—C4	1.4070 (13)	C16—C17	1.3970 (13)
C3—H3A	0.9300	C16—H16A	0.9300
C4—C5	1.3901 (12)	C17—C18	1.3882 (14)
C4—H4A	0.9300	C17—H17A	0.9300
C5—C6	1.3987 (11)	C18—C19	1.3944 (14)
C5—H5A	0.9300	C18—H18A	0.9300
C7—C8	1.4763 (10)	C19—C20	1.3911 (11)
C8—C9	1.3985 (10)	C20—H20A	0.9300
C8—C13	1.3995 (11)		
			
C10—O1—H1O1	110.5 (11)	C9—C10—C11	120.33 (7)
C19—O2—H1O2	113.3 (10)	C12—C11—C10	119.59 (7)
C7—N1—C1	106.91 (6)	C12—C11—H11A	120.2
C7—N1—C14	129.07 (7)	C10—C11—H11A	120.2
C1—N1—C14	123.56 (7)	C11—C12—C13	120.69 (8)
C7—N2—C6	105.62 (7)	C11—C12—H12A	119.7
N1—C1—C2	131.58 (8)	C13—C12—H12A	119.7
N1—C1—C6	105.82 (7)	C12—C13—C8	119.56 (7)
C2—C1—C6	122.56 (7)	C12—C13—H13A	120.2
C3—C2—C1	116.37 (8)	C8—C13—H13A	120.2
C3—C2—H2A	121.8	N1—C14—C15	112.50 (6)
C1—C2—H2A	121.8	N1—C14—H14A	109.1
C2—C3—C4	121.56 (8)	C15—C14—H14A	109.1
C2—C3—H3A	119.2	N1—C14—H14B	109.1
C4—C3—H3A	119.2	C15—C14—H14B	109.1
C5—C4—C3	121.52 (8)	H14A—C14—H14B	107.8
C5—C4—H4A	119.2	C16—C15—C20	119.87 (7)
C3—C4—H4A	119.2	C16—C15—C14	120.66 (7)
C4—C5—C6	117.39 (8)	C20—C15—C14	119.46 (7)
C4—C5—H5A	121.3	C15—C16—C17	119.85 (9)
C6—C5—H5A	121.3	C15—C16—H16A	120.1
N2—C6—C1	109.41 (7)	C17—C16—H16A	120.1
N2—C6—C5	130.08 (8)	C18—C17—C16	120.42 (9)
C1—C6—C5	120.50 (7)	C18—C17—H17A	119.8
N2—C7—N1	112.19 (7)	C16—C17—H17A	119.8
N2—C7—C8	124.10 (7)	C17—C18—C19	119.60 (8)
N1—C7—C8	123.52 (7)	C17—C18—H18A	120.2
C9—C8—C13	120.26 (7)	C19—C18—H18A	120.2
C9—C8—C7	121.37 (7)	O2—C19—C20	121.29 (8)
C13—C8—C7	118.31 (7)	O2—C19—C18	118.63 (8)
C10—C9—C8	119.55 (7)	C20—C19—C18	120.08 (8)
C10—C9—H9A	120.2	C19—C20—C15	120.13 (8)
C8—C9—H9A	120.2	C19—C20—H20A	119.9
O1—C10—C9	117.03 (7)	C15—C20—H20A	119.9
O1—C10—C11	122.64 (7)		
			
C7—N1—C1—C2	175.17 (8)	N1—C7—C8—C13	121.97 (8)
C14—N1—C1—C2	2.30 (12)	C13—C8—C9—C10	−1.55 (12)
C7—N1—C1—C6	−2.40 (8)	C7—C8—C9—C10	175.65 (7)
C14—N1—C1—C6	−175.27 (6)	C8—C9—C10—O1	−178.61 (7)
N1—C1—C2—C3	−178.27 (8)	C8—C9—C10—C11	0.81 (12)
C6—C1—C2—C3	−1.05 (11)	O1—C10—C11—C12	179.82 (8)
C1—C2—C3—C4	−1.54 (12)	C9—C10—C11—C12	0.44 (13)
C2—C3—C4—C5	1.89 (13)	C10—C11—C12—C13	−0.95 (13)
C3—C4—C5—C6	0.40 (12)	C11—C12—C13—C8	0.22 (13)
C7—N2—C6—C1	−1.16 (8)	C9—C8—C13—C12	1.04 (12)
C7—N2—C6—C5	180.00 (8)	C7—C8—C13—C12	−176.24 (8)
N1—C1—C6—N2	2.23 (8)	C7—N1—C14—C15	−98.85 (9)
C2—C1—C6—N2	−175.61 (7)	C1—N1—C14—C15	72.35 (9)
N1—C1—C6—C5	−178.80 (7)	N1—C14—C15—C16	−121.88 (9)
C2—C1—C6—C5	3.37 (11)	N1—C14—C15—C20	59.08 (10)
C4—C5—C6—N2	175.81 (8)	C20—C15—C16—C17	0.98 (14)
C4—C5—C6—C1	−2.93 (11)	C14—C15—C16—C17	−178.05 (9)
C6—N2—C7—N1	−0.41 (8)	C15—C16—C17—C18	−1.43 (17)
C6—N2—C7—C8	174.77 (7)	C16—C17—C18—C19	0.02 (18)
C1—N1—C7—N2	1.81 (8)	C17—C18—C19—O2	−178.72 (10)
C14—N1—C7—N2	174.16 (7)	C17—C18—C19—C20	1.83 (16)
C1—N1—C7—C8	−173.40 (7)	O2—C19—C20—C15	178.29 (8)
C14—N1—C7—C8	−1.05 (11)	C18—C19—C20—C15	−2.28 (14)
N2—C7—C8—C9	130.08 (8)	C16—C15—C20—C19	0.86 (13)
N1—C7—C8—C9	−55.28 (11)	C14—C15—C20—C19	179.91 (8)
N2—C7—C8—C13	−52.67 (10)		

### Hydrogen-bond geometry (Å, °)

**Table:**

*D*—H···*A*	*D*—H	H···*A*	*D*···*A*	*D*—H···*A*
O1—H1O1···O2^i^	0.931 (19)	1.712 (19)	2.6406 (9)	175.4 (17)
O2—H1O2···N2^ii^	0.994 (19)	1.646 (19)	2.6297 (10)	169.7 (16)
C3—H3A···O1^iii^	0.93	2.57	3.2987 (10)	136
C9—H9A···O1^iv^	0.93	2.59	3.4287 (10)	150
C12—H12A···Cg1^ii^	0.93	2.67	3.4521 (9)	142

Symmetry codes: (i) *x*+1/2, −*y*+3/2, *z*−1/2; (ii) −*x*+1, −*y*+1, −*z*+1; (iii) *x*−1/2, −*y*+1/2, *z*+1/2; (iv) −*x*+1, −*y*+1, −*z*.
